# Non-steroidal anti-inflammatory drugs, Cyclooxygenase-2 inhibitors and paracetamol use in Queensland and in the whole of Australia

**DOI:** 10.1186/1472-6963-8-196

**Published:** 2008-09-24

**Authors:** Nadia Barozzi, Susan E Tett

**Affiliations:** 1 School of Pharmacy, University of Queensland, Steele Building, Brisbane, QLD 4072, Australia; 2 Faculty of Health Sciences, University of Queensland, Edith Cavell Building, Royal Brisbane and Women's Hospital, Herston, QLD 4029, Australia

## Abstract

**Background:**

Cross national drug utilization studies can provide information about different influences on physician prescribing. This is important for medicines with issues around safety and quality of use, like non selective non-steroidal anti-inflammatory drugs (ns-NSAIDs) and cyclo-oxygenase-2 (COX-2) inhibitors. To enable comparison of prescription medicine use across different jurisdictions with a range of population sizes, data first need to be compared within Australia to understand whether use in a smaller sub-population may be considered as representative of the total use within Australia. The aim of this study was to compare the utilization of non selective NSAID, COX-2 inhibitors and paracetamol between Queensland and Australia.

**Method:**

Dispensing data were obtained for concession beneficiaries for Australia for ns-NSAIDs, COX-2 inhibitors and paracetamol subsidized by the PBS over the period 1997–2003. The same data were purchased for Queensland. Data were converted to Defined Daily Dose (DDD)/1000 beneficiaries/day (World Health Organization anatomical therapeutic chemical classification, 2005).

**Results:**

Total NSAID and paracetamol consumption were similar in Australia and Queensland. Ns-NSAID use decreased sharply with the introduction of COX-2 inhibitors (from approximately 80 to 40 DDD/1000 beneficiaries/day). Paracetamol was constant (approximately 45 DDD/1000 beneficiaries/day). COX-2 inhibitors consumption was initially higher in Queensland than in the whole of Australia.

**Conclusion:**

Despite initial divergence in celecoxib use between Queensland and Australia, the use of ns-NSAIDs, COX-2 inhibitors and paracetamol overall, in concession beneficiaries, was comparable in Australia and Queensland.

## Background

International comparisons of drug use provide benchmarking data and represent a valuable strategy to achieve a better understanding of use of medicines. Cross national drug utilization studies can provide information on differences in the effect of access to drug programs, the effect of formulary policies and the influences on physician prescribing (education interventions, industry and marketing efforts) to explain some of the prescribing variations. Exploration of the similarities and differences in use is a helpful strategy for planning and improving practices in drug approval, regulation, financing, reimbursement, prescribing and use by patients. In the European Union for example, Eurodurg , was created to promote the drug utilization research across Europe. In Australia the National Prescribing Service (NPS) has used various techniques to improve drug prescribing and use by patients, including academic detailing, audit and feedback and social marketing. However, little research has been done comparing Australian drug use with that of other countries. According to the World Health Organization (WHO) "*the Australian health system is world-class in both its effectiveness and efficiency: Australia consistently ranks in the best performing group of countries for healthy life expectancy and health expenditure per person*.' (World Health Organization 2003). Hence, exploring similarities and differences with the Australian drug utilization patterns may be a valuable opportunity for other countries for planning and developing new ideas for pharmaceutical policy or for education. Reciprocally, the Australian healthcare scheme may further benefit from international comparison study outcomes.

Queensland, one state in Australia, has been shown in a previous study on antilipemic drugs to be suitable for international comparison purposes (to Nova Scotia, one province in Canada) for that group of medicines [1]. Queensland was shown to have a population of similar gender and age distribution, and similar health risk factor exposures, to Nova Scotia. Comparing drug use in Queensland and Nova Scotia has produced very interesting comparisons and contrasts for the statin drugs [1]. These data have provided some useful ideas for changes to influence potentially inappropriate use of those medicines, and also demonstrated that such comparisons can be done between Nova Scotia and Queensland. However, there are advantages in being able to compare to Australia-wide prescription drug use, rather than use in individual states. Accessing data from the individual states in Australia is expensive and data are purchased often with long time delay. On the other hand, accessing Australian data (for the whole country) is rapid and inexpensive. Australian data are freely available on the Medicare Australia website  and the database is updated each month.

The non-selective non-steroidal anti-inflammatory drugs (ns-NSAIDs) are a class of drug used widely [2]. NSAIDs are one of the classes of drugs most prescribed worldwide [2]. However, concern over the overall NSAID consumption has arisen due to issues around their toxicity [2] and also because of their high patterns of utilisation, often in inappropriate population groups [3].

NSAIDs have been used to decrease pain and inflammation for major and minor musculoskeletal disorders for years [4-6]. The most common adverse effect associated with ns-NSAIDs depends on the regulation of the protective mechanisms involving the GI mucosa. GI adverse effects to Ns-NSAIDs were originally proposed to be related to inhibition of one type of enzyme, cyclo-oxygenase-1 (COX-1), and specific drugs (COX-2 inhibitors) were developed to inhibit mainly the 'inducible' cyclo-oxygenase associated with inflammation, cyclo-oxygenase-2 (COX-2) [7]. In 2000, 2001, and 2002 celecoxib, rofecoxib and meloxicam, respectively, three COX-2 inhibitors, were made available in Australia. COX-2 inhibitors were introduced with the recommendation to limit their use to those patients at high risk of GI complications, to those not responding to traditional ns-NSAID therapy or with concomitant use of corticosteroids, anticoagulants and advanced age [8]. However, immediately after their launch on the market a very rapid uptake of use was observed in different countries suggesting that prescribing of those medicines has been excessive [9-16].

The need to improve the safe, appropriate cost effective prescribing of NSAIDs has been recognized in different countries [17,18]. As a consequence, a number of strategies have been planned and implemented to improve and optimize NSAID utilization [17,18]. In 2002, the Australian Government designated arthritis and musculoskeletal conditions as a National Health Priority Area with the aim of improving quality of life for people with those diseases [17]. In 2003, the NPS started an educational program known as "*Optimizing safe and effective use of analgesics in musculoskeletal pain*" and in August 2006, an academic detailing program on the same topic was repeated ("*Analgesic choices in persistent pain, focusing on best practice analgesic treatment in persistent, non-cancer pain*").

The aim of this study was to compare the utilization of ns-NSAIDs, COX-2 inhibitors and paracetamol, in seniors and welfare recipients, between Queensland and Australia in order to determine whether the sub-group, Queensland, was representative of total Australian NSAID utilization over the period when COX-2 inhibitors were introduced into Australia. For future planned international studies of NSAID utilization patterns comparison to the whole of Australia data would be quicker (internet access), cheaper (no cost for Australia collated data) and easier than having to purchase and wait for individual State data.

## Method

Medicare Australia is the agency responsible for payment to community pharmacists for prescription medicines reimbursed by the Pharmaceutical Benefit Scheme (PBS). The PBS subsidizes specific prescription medicines for all Australian residents (national, universal scheme). All the reimbursable prescribed drugs dispensed are recorded in a database, with aggregated, de-identified data publicly accessible through a website [19]. The data are presented aggregated by item number (a code given to each formulation of each compound for medicines subsidized by the PBS). There are two classes of PBS beneficiaries, general and concession. Concession beneficiaries consist of those Australian residents eligible for the Commonwealth Seniors Health Card, Health Care Card and Pensioner Concession Card. These are people receiving old age or disability pensions, single parents, low income families and other patients eligible for a social security benefit. They contribute a low co-payment (currently AUD$4.90), general beneficiaries contribute with higher co-payments (currently AUD$30.70).

The numbers of prescriptions dispensed during the period January 1997 and December 2003, for the whole Australia, for concession beneficiaries for non-selective non-steroidal anti-inflammatory drugs (ns-NSAIDs), COX-2 inhibitors (celecoxib, rofecoxib and meloxicam) and paracetamol were downloaded from the Medicare Australia website . The same data for Queensland were purchased by request from Medicare Australia.

The numbers of eligible concession beneficiaries for each month between January 1997 and December 2003 were obtained by request from Centrelink (the government agency responsible for social services for the Australian community). Australian over 65 years of age are the main beneficiaries of the PBS, responsible for the highest drug consumption [20]. It has been estimated that concession beneficiaries receive approximately 80% of all the PBS subsidized medications. [20]. Moreover, they are the group with highest prevalence of musculoskeletal disorders and most likely to be prescribed the drugs of interest [21]. Conditions approved in Australia for reimbursement of NSAIDs and paracetamol are chronic arthropathies, persistent pain associated with osteoarthritis, symptomatic treatment of osteoarthritis, and symptomatic treatment of rheumatoid arthritis [22].

The generic compounds were classified according to the World Health Organization (WHO) Anatomical Therapeutic Chemical (ATC) classification system 2005 [23]. The ATC M01A, N02BA01 (aspirin), N02BA11 (diflunisal) and N02BE01 (paracetamol) included all the drugs for this study. The number of defined daily dose (DDD) per 1000 concession beneficiaries per day were calculated (for each year) as follows:

1. Total annual consumption of the drug in grams was first calculated and then converted into total DDDs using the following equation:

• Total grams = (N * M * Q)

Where N is the number of PBS services for that item reimbursed; M is the strength of each unit (eg. tablet, capsule etc) and Q is the quantity of units in each service (package size).

• Total DDDs = Total grams/DDD_WHO_

Where DDD_WHO _is the DDD as designated by the WHO (2005).

2. The number of DDDs was then divided by the concession card holder population numbers and the number of days. Hence, drug utilization was expressed as DDD/1000 concession beneficiaries/day and calculated as follows:

DDD/1000 concession beneficiaries/day = total DDDs/(no. of concession beneficiaries/1000 × 365).

The mean DDD/1000 beneficiaries/day for all NSAIDs and paracetamol was compared between Queensland and in the whole of Australia.

DDDs calculations were completed using Microsoft Office Excel 2003 (Microsoft Corporation, Redmond, Washington) and statistical analysis was performed with Origin 6.0 (OriginLab Corporation, Northampton, MA USA). Differences were considered statistically significant if P < 0.05.

Drug Utilization 90% (DU_90%_) for all NSAIDs was also calculated [24]. DU_90% _includes those drugs accounting for 90% of prescriptions within the group of medicines being studied. Data were first expressed as DDD/1000 beneficiaries/day, then percentage of utilization of the total NSAIDs was calculated for each drug. The DU90% comprises those most used, those which summed to 90% of the total consumption [24].

Low-, moderate-, and high-risk of gastrointestinal side effect groups were identified for the ns-NSAIDs as defined by the Gastroenterological Society of Australia [25]. This classification was in agreement with other sources in the literature [26-28]. Ibuprofen and diclofenac were classified as low risk; aspirin, sulindac, naproxen and indomethacin as moderate risk; and ketoprofen and piroxicam as high risk for GI side effects.

## Results

General demographic data, health status data, utilization of health care services and risk factors were similar when comparing Queensland (subset of national population) to the whole of Australia (Table 1). The population in Australia and Queensland were respectively 20,091,500 and 3,888,077 at June 2004 [29]. In both geographical areas concession beneficiaries cards holder accounted for 24.7% of the population (4,963,281 in Australia and 958,405 in Queensland).

**Table 1:** Demographics and relevant health status: Comparison between Queensland and Australia.

**Demographics and relevant health status: comparison between Queensland and Australia**		
	**QLD**	**AUS**

**Demographics and health status**		

*Demographics*		

• Total adults aged 65 and over	12%	13%
• Males aged 65 and over	46%	45%
• Females aged 65 and over	54%	55%
*Health Status – Prevalence of musculoskeletal disease as a long term condition*		
• Total Musculoskeletal disease	34%	32%
◦ Arthritis	14%	14%
◦ Rheumatism	1%	1%
◦ Back pain/problems neck/disc disorders	23%	21%
◦ Osteoporosis	2%	2%
◦ Other	5%	5%

**Utilisation of health care services**		

• Average Medicare services processed per person	11	11
• Average Medicare services processed per person aged ≥ 65	22	23
• Pension Card and Commonwealth Seniors health cards as a proportion of all concessions	60%	64%
*Pharmaceutical Benefit Scheme (services related to the musculoskeletal system)*		
• Average number of services per person	8	8
*Pharmaceutical Benefit Scheme (overall)*		
• Average number of services per person	1	1

**Risk factors**		

• Overweight/obese adults (males aged 18 and over)	56%	54%
• Overweight/obese adults (females aged 18 and over)	41%	38%
• Adults who are physically inactive males aged 18 and over	40%	42%
• Adults who are physically inactive females aged 18 and over	31%	31%

Data extracted from the 2001 Australian Census and Medicare Australia annual report 2000–2001 and 2002–2003 [45]

**Figure 1:**
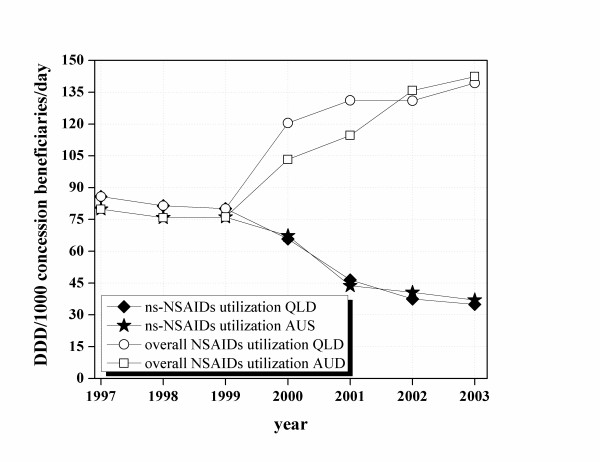
The ns-NSAID and COX-2 inhibitor prescription pattern in concession beneficiaries in Australia and in Queensland between 1997 and 2003.

**Figure 2:**
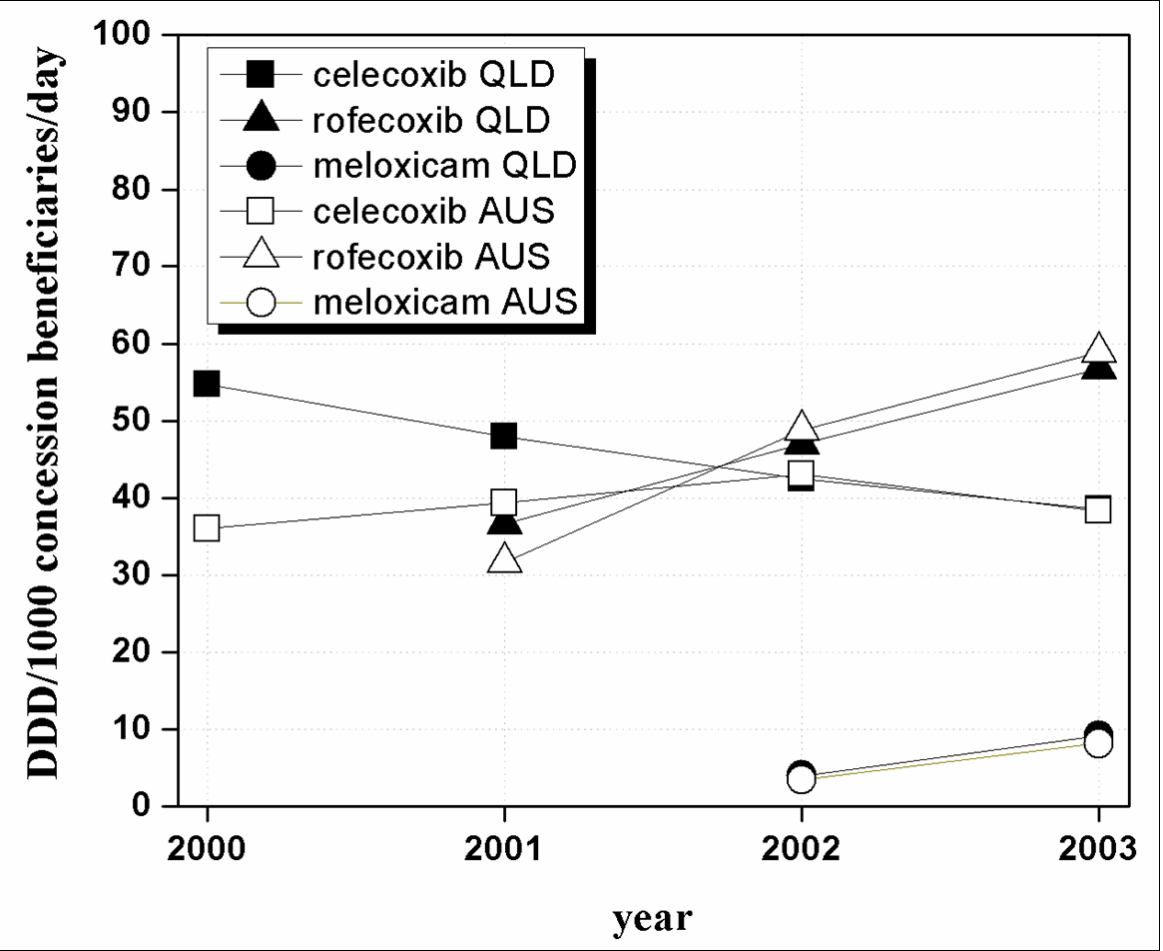
COX-2 inhibitor prescription pattern in concession beneficiaries in Australia and in Queensland between 2000 and 2003.

In Figure 1, ns-NSAIDs and overall NSAIDs dispensing data for concession beneficiaries, over the period 1997–2003, in Australia and Queensland are shown. Ns-NSAID consumption in concession beneficiaries was similar in Australia and in Queensland. Ns-NSAID use decreased sharply with the introduction of COX-2 inhibitors (from approximately 80 to 40 DDD/1000 concession beneficiaries/day) (Figure 1). COX-2 inhibitors uptake was high in the period 2000–2003. The overall use of COX-2 inhibitors started at about 60 and 40 DDD/1000 concession beneficiaries/day, respectively in Queensland and Australia, when celecoxib was introduced into Australia (2000). There was initial different use of COX-2 inhibitors: 55 DDD/1000 concession beneficiaries in Queensland vs 36 DDD/1000 concession beneficiaries in Australia (P = 0.46) (Figure 2). However, as the other COX-2 inhibitors (rofecoxib and meloxicam) came on to the market (2002), use rapidly became similar in Australia and in Queensland (approximately 45 DDD/1000 concession beneficiaries/day) (Figure 2).

Paracetamol consumption was similar in Australia and Queensland and it was constant over the time.

DU90% patterns were similar in Australia and Queensland (Table 2). Major changes were seen in both jurisdictions after the introduction of COX-2 inhibitors. DU90% showed that before the COX-2 inhibitors introduction, naproxen and diclofenac were the most used ns-NSAIDs (1997–1999). When COX-2 inhibitors took over, these ns-NSAIDs were progressively replaced and celecoxib and rofecoxib became the most used anti-inflammatory drugs (2000–2003) (Table 2).

**Table 2:** DU_90% _in Queensland and in Australia for the overall NSAID utilization in Concession beneficiaries between 1997 and 2003

**Australia DU**_90%_				
1997–1999%	2000%	2001%	2002%	2003%

Naproxen* 28	Celecoxib^+ ^35	Celecoxib^+ ^40	Celecoxib^+ ^39	Celecoxib^+ ^35
Diclofenac** 21	Naproxen* 20	Rofecoxib^+ ^16	Rofecoxib^+ ^22	Rofecoxib^+ ^25
Ketoprofen^# ^16	Diclofenac** 15	Naproxen* 16	Naproxen* 12	Diclofenac** 12
Piroxicam^# ^10	Ketoprofen^# ^9	Diclofenac** 14	Diclofenac** 12	Naproxen* 11
Indomethacin* 7	Piroxicam^# ^7	Ketoprofen^# ^7	Ketoprofen^# ^5	Meloxicam^+ ^9
Ibuprofen** 6	Ibuprofen** 5			

**Queensland DU**_90%_				

1997–1999%	2000%	2001%	2002%	2003%

Naproxen* 27	Celecoxib^+ ^45	Celecoxib^+ ^43	Celecoxib^+ ^40	Celecoxib^+ ^35
Diclofenac** 20	Naproxen* 14	Rofecoxib^+ ^16	Rofecoxib^+ ^22	Rofecoxib^+ ^26
Ketoprofen^# ^17	Diclofenac** 13	Naproxen* 10	Diclofenac** 9	Diclofenac** 9
Piroxicam^# ^12	Ketoprofen^# ^8	Diclofenac** 10	Naproxen* 9	Meloxicam^+ ^8
Ibuprofen** 9		Ketoprofen^# ^5	Ibuprofen** 5	Naproxen* 8
Indomethacin* 5		Ibuprofen** 5	Ketoprofen^# ^4	Ibuprofen** 5

^+ ^Cox-2 inhibitors	** ns-NSAIDs low risk	^# ^ns-NSAIDs high risk	* ns-NSAIDs moderate risk	

## Discussion

The data collected in this study indicate that Australian data as a whole, for concession beneficiaries, were comparable to data from Queensland, in terms of ns-NSAIDs, COX-2 inhibitors and paracetamol. Queensland had only about 19% of the population of Australia, so it was not the major 'driver' of use, indicating that this finding was not simply due to measuring drug use in a majority group which subsequently determined overall use in the whole population.

Celecoxib use in Australia and Queensland was initially different when the drug was first listed on the PBS (2000) (Figure 2). However, the difference observed was not statistically significant (P ≥ 0.05). The overall COX-2 inhibitors, and consequently the total NSAID use became essentially the same in Australia and Queensland by 2002 (Figure 1 and 2). Reasons for this initial difference were explored. Evidence was lacking about any specific initiatives which may have made Queensland prescribing of celecoxib any different to that of the rest of Australia, however, possible reasons are interesting to speculate about. Celecoxib was the first COX-2 inhibitor marketed in Australia and approved for listing on the national formulary of reimbursable medications (PBS). Celecoxib was listed as a restricted benefit for symptomatic treatment of osteoarthritis (OA) and rheumatoid arthritis (RA). OA is the most common musculoskeletal condition, affecting more than 1.6 million Australian while RA affects a much smaller group[21]. OA incidence rises with age affecting mainly people over 65 years of age[21]. However, prevalence of disease was similar in Queensland and in the whole Australian population, so this cannot account for any initial differences in COX-2 uptake (Table 1). Also, the older population (over 65 years of age) has been calculated to be very similar in Queensland and in the whole of Australia (13% of the whole Australian population and 12% in Queensland) so this also cannot account for the initial differences seen in celecoxib use [29].

A possible explanation of the differences observed in the celecoxib prescribing initially, could have been attributed to a higher older population in Queensland, compared to the rest of Australia, and consequently, to higher disease prevalence (OA). However, in our study, comparison of selected indicators such as of demographic data, health status data, utilization of health care services and risk factors between Queensland and Australia did not sustain this as an explanation (Table 1). There is the common perception that the older population might be increasing in Queensland because the trend for retirement is to move to coastal areas in Queensland[21,30]. However, socio-economic and demographic data do not support this perception [29]. The number and distribution of retirement homes in Australia also shows no disproportionate distribution [29]. Another explanation investigated was the possibility of heavier marketing activity or more press releases in Queensland. However, despite the fact that some information can be sourced in the literature about celecoxib marketing activity in Australia and this potential influence on prescribing behaviour [10,31], no evidence was found to differentiate marketing strategies used in the different States within Australia. There were also no obvious differences in opinion leaders' influence in the different States, although this can not be discounted as this is not quantified in anyway.

Associations between prescribing behaviour and physician characteristics (age, gender and years from graduation for example) have been described [32,33]. Volume of prescription has been reported to be related to updated knowledge about the relative risks and benefits of drugs with similar indications (ns-NSAIDs versus COX-2 inhibitors or paracetamol in this case) [33]. Younger physician, who have been trained more recently, seemed to have higher familiarity with the benefits emerging from new therapies whereas more experienced prescribers might be more critical about the possible risks [33]. However, in Australia it is not possible to identify the prescriber, and the lack of linked information to the prescribing data did not allow a deeper analysis to determine any association with prescriber characteristics or whether different geographical areas differed in respect to physician characteristics. The lack of linked information was one of the main limitations of the study.

In Australia prescription data are also not linked to other data sources (e.g. medical services or hospitalizations). Consequently, it was not possible to establish from our data if the prescription of any of the drugs was appropriate, if those drugs were prescribed for the correct indications (e.g. correct dosage and/or duration), or if prescriptions came from general practitioners or specialists; neither was it possible to complete any analysis of co-morbidity or co-prescription.

Despite the initial difference in celecoxib use, the introduction of the COX-2 inhibitors led to a progressive increase in total NSAID use whereas paracetamol use remained steady both in Queensland and Australia. It is possible that an increase in patients (concession beneficiaries) treated may have raised the number of NSAID prescriptions. However, prevalence of disease does not appear to have changed over this relatively short time period.

Patterns in drug use can vary from region to region for reasons such as differences in the local guidelines, general cultural background, personal history of health practitioners' (e.g. education received and country of origin), patients' characteristics, and healthcare systems (e.g. reimbursement plans, health policies) [32]. In Australia, pharmaceutical reimbursement plans and health policies are organized at a national level, this means that the same rules for coverage and reimbursement apply to the Queensland and to the overall Australian concession beneficiaries. Moreover, no differences in patients' characteristics (e.g. age and gender, risk factors, general, prevalence of musculoskeletal disease, and utilization of healthcare services) were observed between Queensland and Australia (Table 1).

The first-line therapy recommended for people with chronic pain such as OA is generally paracetamol [31,34-36]. At therapeutic doses, paracetamol is considered safer than other anti-inflammatory drugs and it is much cheaper than ns-NSAIDs and COX-2 inhibitors [37]. However, paracetamol prescribing has been very steady all over the period (1997–2003), both in Queensland and in Australia as a whole, which is a little disappointing considering the potential benefits. Paracetamol is a much safer option for elderly suffering from musculoskeletal conditions. It is possible that concession beneficiaries may have received paracetamol as an adjuvant during NSAID therapy. However, in Australia it is not possible to identify co-prescriptions.

The overall change in drug use can be identified in the DU_90% _profiles (Table 2). DU_90% _was introduced by Bergman et al. as a source for more specific analysis of quality in prescribing [24]. DU_90% _can be used in a number of ways, for example, to look at trends or changes in drug use, as is done here, or on an individual basis to encourage health practitioners to evaluate their prescribing behavior, compared to others, to enable them to review the drugs included in their personal formulary [38]. The DU_90% _for Australia and Queensland were very similar all over the period, showing the marked change from ns-NSAIDs to the predominant use of COX-2 inhibitors.

The prescription patterns in this study agreed with data from other studies. Mamdani et al. showed that in their population cohort (also elderly aged 66 and over) NSAID use had increased by 41% and the rise was entirely due to the uptake of COX 2 inhibitors [39]. Similarly, before rofecoxib withdrawal, COX-2 inhibitor prescribing increased the overall use of the NSAID class in Scotland, Germany, Norway, Ireland and Portugal [40-44].

The ATC/DDD index chosen for consistent use for this study was the 2005 version. The WHO recommends use of a consistent dated version, rather than changing the DDD mid-way through a study time. However, in 2003 the assigned DDD for rofecoxib had changed from 12.5 mg to 25 mg. Clinical practice had changed during this time, with the more common dosage increasing. Therefore applying the later DDD (25 mg) to earlier than 2003 data could have led to an underestimate in the actual number of DDD used. For the early years, therefore, use of rofecoxib may have actually been higher than that reported here, due to an artifact of the changing DDD and changing clinical practice. However, for the purposes of the present study this would not alter any conclusions, nor any of the comparisons described here, as the same artifact would be present for the Queensland state data as for all the Australian data.

In conclusion, this study showed that despite the initial divergences in celecoxib use between Queensland and Australia, the use of ns-NSAIDs, COX-2 inhibitors and paracetamol overall, in concession beneficiaries, was comparable in Australia and Queensland. The differences observed were only temporary, when this new class of drugs was first introduced. Both jurisdictions demonstrate an overall increase in NSAID use following the introduction of COX-2 inhibitors. Australian data may be a valid alternative to Queensland data for future international comparison studies on ns-NSAIDs and COX-2 inhibitors utilization. By using Australian data time delays and costs will decrease, facilitating the process of data collection.

## Pre-publication history

The pre-publication history for this paper can be accessed here:


